# Phase Transformations During Softening of Iron Ore Sinter of Varying Basicity in the CaO–SiO_2_–FeO System

**DOI:** 10.3390/ma19102034

**Published:** 2026-05-13

**Authors:** Elena A. Vyaznikova, Andrey N. Dmitriev, Galina Yu. Vitkina, Vladimir V. Katayev

**Affiliations:** Vatolin Institute of Metallurgy of the Ural Branch of the Russian Academy of Sciences, 620016 Ekaterinburg, Russia; vjaznikova@mail.ru (E.A.V.); andrey.dmitriev@mail.ru (A.N.D.); kataev.5959@mail.ru (V.V.K.)

**Keywords:** blast furnace, iron ore sinter, softening, phase composition, SFCA, wustite, basicity, reduction

## Abstract

The cohesion zone of a blast furnace is instrumental in determining the gas-dynamic regime and the efficiency of reducing gas utilization. The extent of this phenomenon is contingent upon the initial and final temperatures at which iron ore undergoes softening, which, in turn, are determined by the chemical and phase composition, as well as the degree of reduction of the charge. The present study investigated sinter with a basicity (CaO/SiO_2_) ranging from 1.2 to 3.0 using a combination of methods. The experimental program involved the use of X-ray diffraction (XRD) with refinement using the Rietveld method, scanning electron microscopy with energy-dispersive X-ray spectroscopy (SEM-EDS), and load-dependent softening tests. It was established that as the basicity increased, the content of the calcium–aluminum silicoferrite (SFCA) binder phase increased from 6.2 to 17.5 wt.%, whilst the amount of hematite decreased from 12.6 to 2.3 wt.%. The softening onset temperature increases from 1185 to 1260 °C, the softening end temperature from 1345 to 1415 °C, and the softening interval narrows from 160 to 155 °C. The evolution of the phase composition of sinter during controlled reduction (0–95%) has been investigated for the first time. It has been demonstrated that the maximum accumulation of wustite (FeO) is attained at a reduction degree of 40–60%, irrespective of the basicity of the substance. It is precisely in this range that the minimum softening start (1040–1065 °C) and end (1170–1210 °C) temperatures are observed, which is associated with the formation of low-melting eutectics. The sinter belongs to the CaO–SiO_2_–FeO–Al_2_O_3_–MgO system, and the softening behavior is governed by the FeO–CaO–SiO_2_ system where low-melting eutectics form. When the reduction rate exceeds 60%, the metallic phase becomes dominant, leading to an increase in softening temperatures and a narrowing of the cohesion zone. It is evident from the data obtained that the optimal basicity range of the sinter is 2.0–2.5. Furthermore, it is recommended that a reduction degree of at least 60% is implemented in order to improve gas dynamics and increase blast furnace productivity. The findings can be utilized to enhance the efficiency of charge materials and refine mathematical models of the blast furnace process.

## 1. Introduction

In view of the present paucity of high-quality iron ore and the progressively stringent environmental requirements for the blast furnace process, the most critical task is to enhance the efficiency of reducing gas utilization and minimizing coke consumption [1,2,3]. One of the factors determining the gas-dynamic regime and thermal performance of a blast furnace is the state of the cohesion zone. This is the region where charge materials are in a visco-plastic state and undergo softening and melting [4,5,6]. The length of the cohesion zone is contingent on the softening interval of the iron ore materials: the narrower this interval and the lower the zone is located, the more effective the gas permeability of the charge column and the higher the efficiency of utilizing the gas’s reducing potential [7,8].

In the context of iron ore materials, sinter assumes a predominant role, accounting for an average of 50 to 80% of the charge in contemporary blast furnaces [9]. The behavior of the material under investigation at elevated temperatures is determined by its chemical and phase composition, which, in turn, are contingent on basicity (CaO/SiO_2_) and sintering conditions [10,11,12]. As the level of basicity rises, the proportion of the binding phase—calcium and aluminum silicoferrites (SFCA)—rises concomitantly, thereby ensuring the sinter’s high strength and reducing capacity [13,14,15]. However, under the conditions of blast furnace smelting, as the charge moves downward and the temperature rises, iron oxides undergo reduction and phase transformations that can alter the softening and melting temperature ranges [16,17].

It has been demonstrated by numerous studies that the onset and end temperatures of softening are contingent not solely on the basicity and mineral composition of the initial sinter, but also on its degree of reduction [18,19,20]. Of particular importance is the reduction range of 40–60%, corresponding to the maximum accumulation of wustite (FeO) in the sinter. The presence of wustite, in conjunction with the silicate component, results in the formation of low-melting eutectics. This phenomenon leads to a reduction in softening temperatures and an augmentation of the cohesion zone [21,22,23]. Further reduction (>60%) of iron results in the conversion of the element to its metallic form, accompanied by a substantial increase in melting temperatures. This phenomenon contributes to a downward shift in the cohesion zone [24,25].

Recent advances in sintering technology, such as the super-high bed homogeneous sintering process, have demonstrated that achieving uniform sinter quality is critical for stable blast furnace operation and carbon emission reduction [26]. Xu et al. showed that homogenizing the liquid phase formation during sintering dramatically improves the mechanical strength and reducibility of the product, which directly affects its high-temperature performance [26]. These findings underscore the importance of understanding the intrinsic softening and phase transformation behavior of sinter under controlled conditions.

Despite the significant number of studies devoted to the mineralogy of sintered bodies [27,28,29,30], a systematic analysis of phase transformations over a wide range of basicity (from 1.2 to 3.0) in combination with controlled reduction and quantitative assessment of phase composition is still lacking. In particular, there are very few studies in which the Ritveld method has been used to quantitatively assess the content of the SFCA phase and its transformation during reduction, and to establish a relationship between these changes and softening characteristics [31,32]. The majority of studies are confined to specific values of basicity or do not take into account the influence of the degree of reduction on phase composition.

The objective of this study is to methodically examine the phase composition and microstructure of agglomerates exhibiting basicity levels ranging from 1.2 to 3.0, with a view of ascertaining the softening temperature ranges contingent on basicity and the degree of reduction. Furthermore, the study seeks to establish quantitative relationships between phase transformations (notably the decomposition of SFCA and the accumulation of wustite) and alterations in softening temperatures. The results will justify the selection of the optimal basicity in relation to the degree of reduction of the sinter to minimize the cohesion zone and improve the technical and economic performance of blast furnace smelting.

## 2. Materials and Methods

### 2.1. Research Subjects and Chemical Analysis

For the study, sinter with a basicity (mass ratio CaO/SiO_2_) ranging from 1.2 to 3.0 in 0.2 increments were selected. The sampling procedure was conducted in accordance with the stipulated guidelines set out in the Russian State Standard 15054-80 [33] (equivalent ISO 3082:2017 [34]). The chemical composition was determined using titrimetric analysis (major oxides) and SEM-EDS (phase-specific elemental analysis). The errors in titrimetric analysis did not exceed 0.5% relative; EDS provides semi-quantitative data. The results of the study are presented in Table 1. The samples exhibited comparable concentrations of SiO_2_ (4.95–5.06 wt.%), MgO (1.77–2.40 wt.%), and Al_2_O_3_ (0.55–0.64 wt.%), thereby enabling the assessment of the influence of basicity to be conducted without significant interference from other components. FeO is listed separately because it is the main phase controlling the formation of low-melting eutectics in the FeO–CaO–SiO_2_ system during reduction.

### 2.2. X-Ray Diffraction Analysis

The phase compositions of the initial sinter and samples after partial reduction were determined using a Shimadzu XRD-7000 X-ray diffractometer, Shimadzu Corporation, Tokyo, Japan (operating under CuKα radiation, with a tube voltage of 22 kV and a current of 30 mA). The measurements were conducted at ambient temperature in the 2θ range of 10–85°, at a scanning rate of 1°/min. The procedure for sample preparation involved grinding to a particle size of less than 0.063 mm. Quantitative phase analysis was performed using the Rietveld method with the Topas 6.0 software package (Bruker AXS GmbH, Karlsruhe, Germany) and the PDF-4+ database of the International Centre for Diffraction Data (ICDD). For each degree of reduction, a minimum of two parallel samples were analyzed; the phase determination error did not exceed 2–3 wt.%.

### 2.3. Reduction of Sinter

A laboratory vertical detachable tube furnace (JSC “Aramil Plant of advanced technologies” (JSC “AZPT”), Aramil, Russia) with a gas-tight retort, equipped with a mass-flow controller for CO/N_2_, was utilized to prepare samples exhibiting varying degrees of reduction (0, 5, 10, 20, 40, 60, 80, 95%). Sinter samples, in the form of pieces measuring 10–12.5 mm, were placed in a reaction chamber and heated to 900 °C in a nitrogen atmosphere at a flow rate of 5 L/min. The samples were then held at this temperature for 30 min, with the gas flow rate increased to 15 L/min. Following this, a CO/N_2_ (30/70 vol.%) reducing mixture was introduced at the same flow rate, while the sample’s mass loss during reduction was continuously monitored.

The second reduction method entailed the simultaneous heating of sinter samples ranging in size from 3 to 5 mm with pulverized coal of 1.25 mm particle size in a 2:1 ratio in a nitrogen atmosphere. The compacted mixture was heated at a rate of 10 °C/min until a set temperature was reached, after which it was cooled with a constant nitrogen purge at a rate of 1 L/min. This second method was used to simulate pre-reduction of sinter by carbonaceous materials before the softening zone.

The extent of reduction was determined by measuring the mass loss and calculating the ratio of removed oxygen to the total oxygen content in the iron oxides. Upon attaining the targeted degree of reduction, the samples were subjected to cooling in a nitrogen atmosphere to room temperature, a process designed to avert oxidation.

The degree of reduction (*R*) was calculated with the following formula:

$$R=\left(\frac{0.111\cdot FeO^{\prime }+0.43{\cdot Fe^{\prime }}_{met}}{0.43\cdot {Fe^{\prime }}_{total}}+\frac{\left(m^{\prime }-m\right)\cdot 100}{m^{\prime }\cdot 0.43\cdot {Fe^{\prime }}_{total}}\right)\cdot 100\;,$$

where *R* is the degree of reduction, %; *FeO′* is the mass fraction of wustite (before reduction), %; *Fe′_met_* is the mass fraction of metallic iron (before reduction), %; *Fe′_total_* is the mass fraction of total iron (before reduction), %; *m′* is the mass of the material (before reduction), g; *m* is the mass of the material (after reduction), g.

Each reduction experiment was repeated three times; the standard deviation in the final reduction degree was within ±2%.

### 2.4. Determination of Softening Temperatures

The initial and final softening temperatures were determined in accordance with the Russian State Standard 26517-85 “Iron ores, agglomerates and pellets. Method for determining the temperature of the beginning of softening and the temperature range of softening” [35]. The experimental apparatus comprised a heating furnace and a loading mechanism. The crucible, which was cylindrical in shape and composed of refractory materials, measured 50 mm in height and 25 mm in diameter. It was filled with sintered material composed of particles ranging in size from 3 to 5 mm. The crucible was then placed within a resistance furnace, and a rod with a load of 0.1 MPa was mounted on top. The heating was conducted at a rate of 10 °C/min in an atmosphere of nitrogen. The softening start temperature was defined as the temperature at which the rod penetrated the sample by 1% of its initial height, and the softening end temperature was defined as the temperature at which the rod penetrated the sample by 40%. Multiple parallel tests were conducted for each sample, with a maximum deviation of 10 °C recorded.

### 2.5. Microstructural Analysis

The microstructure of the original and reduced sinter was examined using a JEOL JSM-5900LV scanning electron microscope (SEM), JEOL Ltd., Tokyo, Japan, equipped with an energy-dispersive microanalyzer (EDS), Oxford Instruments plc, Abingdon, UK. The samples were embedded in epoxy resin, ground, and polished using diamond pastes, then coated with a carbon conductive layer. The analysis was conducted at an accelerating voltage of 20 kV. Quantitative elemental analysis was conducted utilizing standards from SPI Supplies, SPI Supplies/Structure Probe Inc., West Chester, PA, USA. Furthermore, the microstructure was examined using an Olympus GX-51 optical microscope, Olympus Corporation, Tokyo, Japan, for qualitative assessment of phase distribution (quantitative analysis was performed by XRD).

## 3. Results and Discussion

### 3.1. Phase Composition of the Initial Sinter

**Figure 1:**
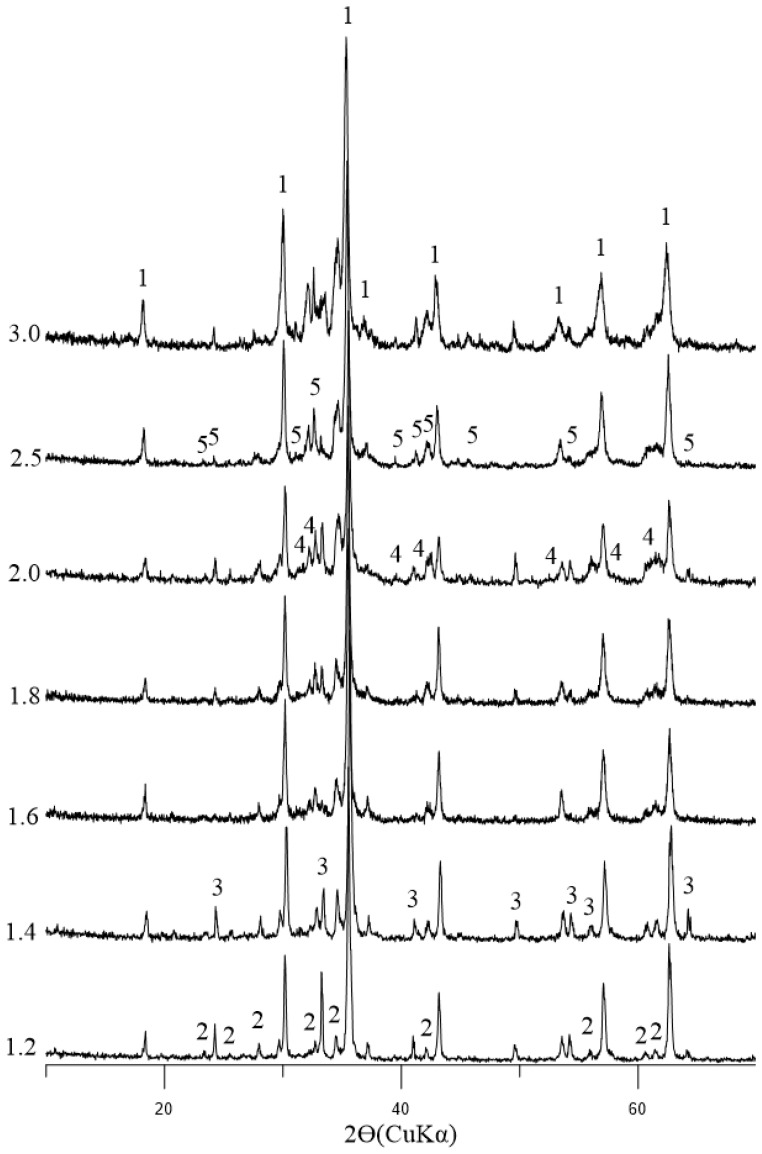
Changes in the phase composition of sinter with increasing basicity. Symbols: 1—Fe_3_O_4_ [01-089-0950], 2—SFCA [00-046-0037], 3—Fe_2_O_3_ [01-071-5088], 4—β-2CaO·SiO_2_ [01-086-0398], 5—α-2CaO·SiO_2_ [01-080-4708]. Experimental errors are within 2–3%.

The results of the study on changes in the phase composition of the initial sinter as the basicity increased from 1.2 to 3.0, based on X-ray phase analysis, are shown in Figure 1; their quantitative compositions are presented in Table 2 (quantitative phase contents remain accurate within 2–3 wt.%).

Table 2 shows only the crystalline phases determined by X-ray phase analysis. The silicate binder of the agglomerates contains a glassy phase that cannot be detected by the standard Rietveld method without the use of an internal standard. The amount of the X-ray amorphous phase was determined separately using the internal standard method (Si), and it was up to 20%. However, the focus of this study was on the changes in the crystalline iron-containing phases (Fe_3_O_4_, Fe_2_O_3_, FeO, SFCA) which determine the softening process. The presence of the amorphous phase is indirectly taken into account when interpreting the results as part of the silicate binder.

The primary iron-bearing phases are magnetite (Fe_3_O_4_) and hematite (Fe_2_O_3_). As the level of basicity rises, the amount of magnetite increases marginally (from 73.6% to 76.2% by mass), while the hematite content decreases from 12.6% to 2.3% by mass. This phenomenon can be attributed to the observation that an elevated CaO content results in the formation of a greater quantity of liquid phase during the sintering process. This, in turn, promotes the growth of magnetite grains and the incorporation of iron into complex ferrites [10,12].

The silicate component consists of various polymorphic modifications of dicalcium silicate (β-, α-, and γ-2CaO·SiO_2_), as well as a bridging phase—calcium–aluminum silicoferrite (SFCA)—with variable composition, but corresponding to the general formula M_14_O_20_, where M is Ca, Fe, Si, Al, or Ti. The SFCA content increases systematically from 6.2 wt.% (basicity 1.2) to 17.5 wt.% (basicity 3.0), a finding that is consistent with the data presented in [13,15,28]. The elevated SFCA content present within highly basic sintered bodies ensures a robust bond between magnetite grains, thereby exerting a favorable influence on low-temperature reducibility [14,32].

### 3.2. The Effect of Basicity on Softening Temperatures

**Figure 2:**
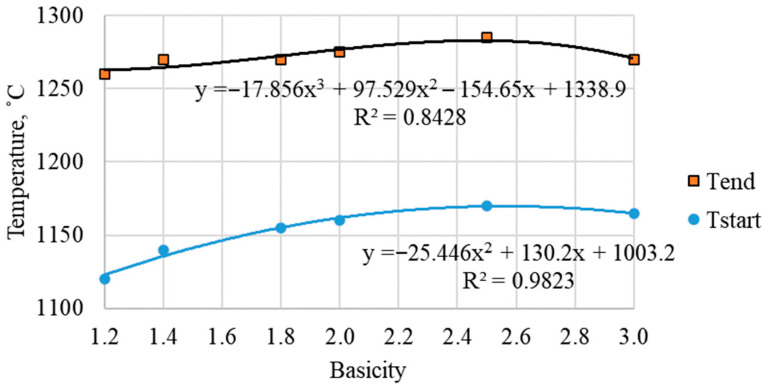
Temperatures at the start and end of sinter softening as a function of basicity (error bars represent the maximum deviation of ±8 °C from parallel softening tests).

As illustrated in Figure 2, a clear correlation exists between the onset and end softening temperatures of sinter and their basicity as the mass ratio of CaO/SiO_2_. As the degree of basicity increases from 1.2 to 3.0, the softening onset temperature rises from 1185 to 1260 °C, and the softening end temperature rises from 1345 to 1415 °C (error ±8 °C). The softening interval (ΔT = T_end_ − T_start_) decreases from 160 to 155 °C. This behavior is explained by the increase in the proportion of the refractory SFCA phase, which forms a strong framework and increases the viscosity of the liquid phase during melting [5,8,16]. Furthermore, a decline in hematite and an increase in magnetite content have been observed, which also contributes to higher softening temperatures. This is due to the fact that magnetite has a higher solidus temperature in the Fe–O system compared to hematite [7,20].

### 3.3. Changes in Phase Composition During Reduction

**Figure 3:**
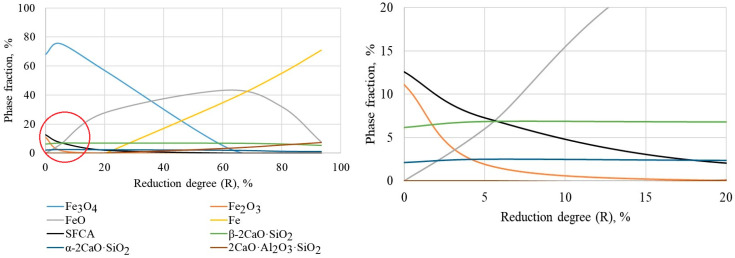
Change in the phase composition of the sinter due to a basicity of 2.0 during reduction (on the right—enlarged image of the graph, bounded by a red circle).

In order to ascertain the factors that precipitate alterations in softening temperatures in the context of blast furnace conditions, a study was conducted on samples of sinter exhibiting varying degrees of reduction (ranging from 0 to 95%). As illustrated in Figure 3, the phase composition of a sinter with a basicity of 2.0 (used here as a representative example) undergoes a specific evolution. In the initial state (0% reduction), magnetite is the predominant constituent of the sinter structure; it contains up to 1.6 wt.% Mg, a small amount of hematite, and silicate phases—SFCA and dicalcium silicate of the β and α modifications. EDS point analyses on clean SFCA grains gave an average approximate composition of Ca_2.3_Mg_0.8_Al_1.5_Fe_8.3_Si_1.2_O_20_. However, EDS is semi-quantitative; typical SFCA compositions reported in the literature vary [10,11,32]. The range of measured Fe content was 7.9–8.7 atoms per formula unit.

**Figure 4:**
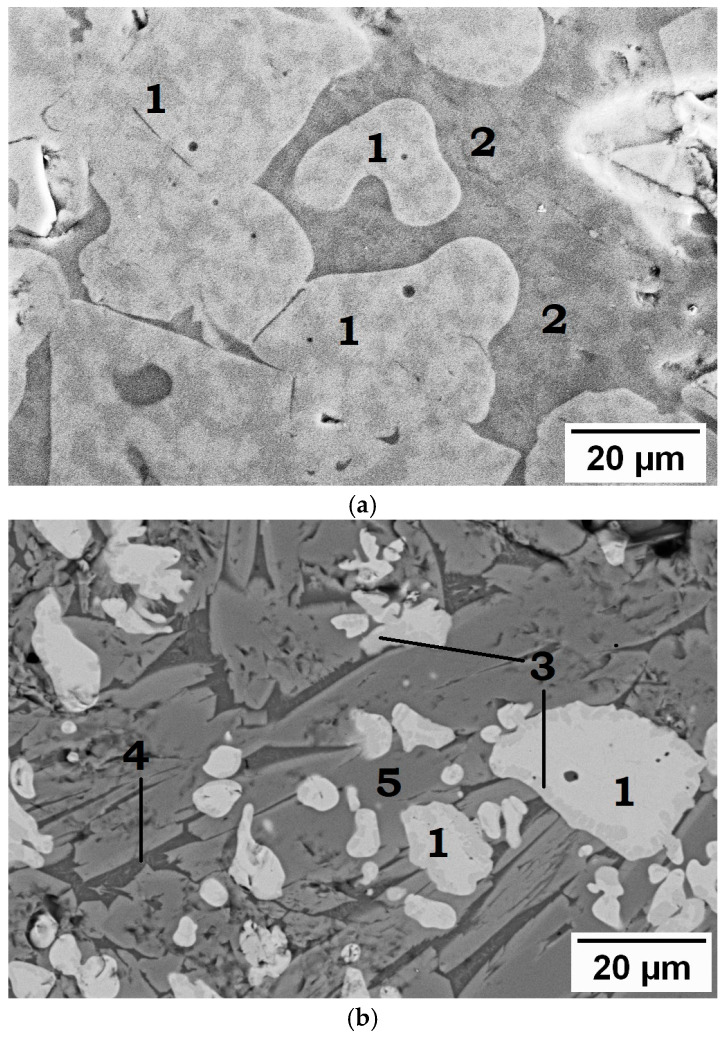

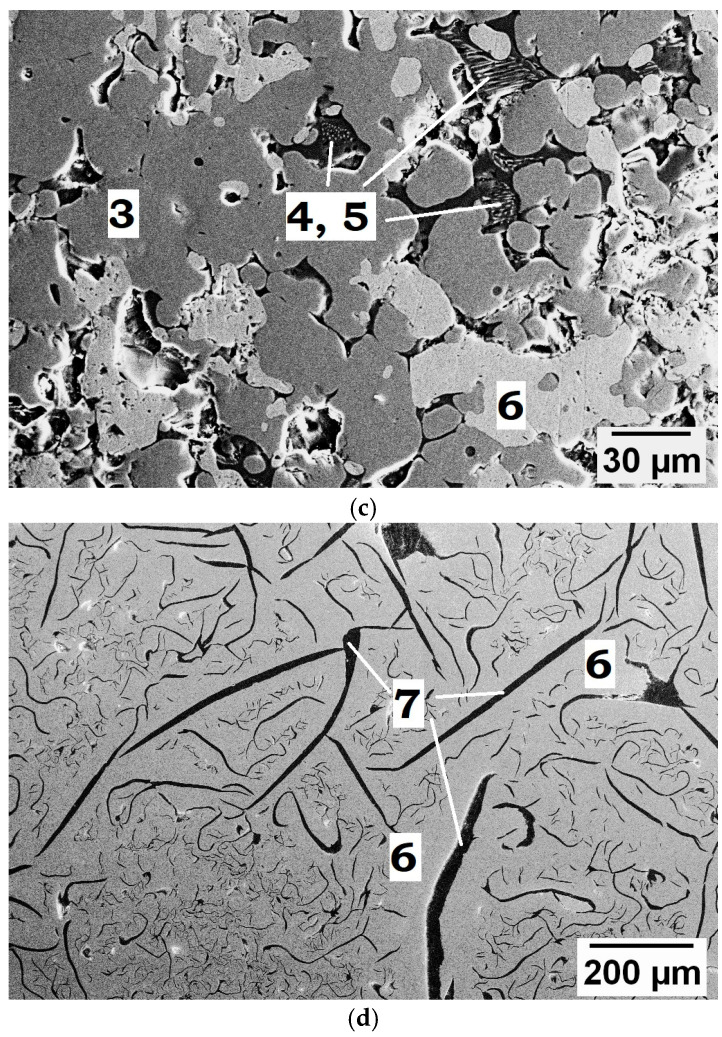
Microstructure of a sinter with a basicity of 2.0 at different degrees of reduction: (**a**) 5%; (**b**) 20%; (**c**) 61%; (**d**) 95%. Symbols: 1—magnetite, 2—SFCA, 3—wustite, 4—gehlenite, 5—merwinite, 6—Fe_met_; 7—graphite.

When the sinter is reduced by 4–7%, hematite is reduced to magnetite, and the decomposition of SFCA begins, resulting in the release of additional magnetite and the formation of calcium silicates (gehlenite Ca_2_Al_2_SiO_7_ and merwinite Ca_3_Mg(SiO_4_)_2_). These data are corroborated by microstructural analyses (Figure 4a).

**Figure 5:**
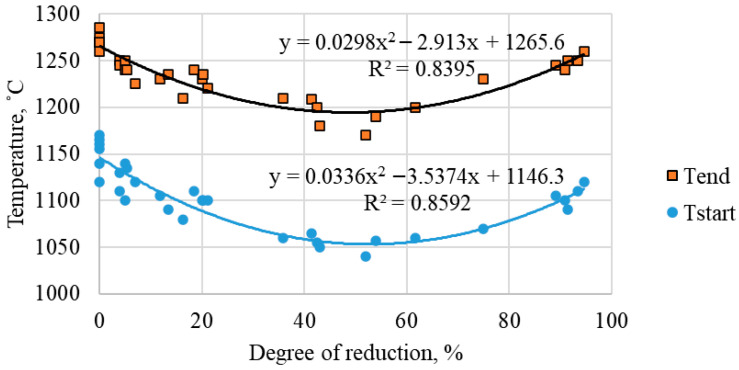
Start and end temperatures of softening as a function of the degree of reduction.

As illustrated in Figure 4b, a reduction degree of 16–21% results in a substantial increase in the amount of wustite (FeO) and almost complete decomposition of SFCA. According to EDS data, wustite is found at the grain boundaries of magnetite (Figure 4b, 3) or in the form of dendritic phases; it contains up to 2.8 wt.% Mg and up to 0.7 wt.% Al, which contributes to its stabilization at high temperatures [21,22]. The binder phase is constituted by a mixture of gehlenite (Figure 4b, 4), merwinite (Figure 4b, 5), and a diopside phase. In this process, an additional amount of dicalcium silicate is formed for low-basic sinter and tricalcium silicate for high-basic sinter.

The maximum accumulation of wustite has been observed to occur at a reduction degree of 40–60% (Figure 4c). In this region, wustite (Figure 4c, 3) becomes the predominant iron-bearing phase, and the first grains of metallic iron appear (Figure 4c, 6). The SFCA phase is not evident in this reduction range. The matrix phase is predominantly composed of gehlenite and merwinite, but it commences partial melting, as demonstrated by the contraction of the samples.

When the sinter is reduced to 74–95% (Figure 4d), further reduction of wustite to metallic iron occurs, along with separation into an iron-bearing phase, represented by cast iron (Figure 4d, 6) with flake graphite (Figure 4d, 7), and a silicate fraction in the form of a mixture of gehlenite Ca_2_Al_2_SiO_7_, merwinite Ca_3_Mg(SiO_4_)_2_, diopside CaMgSi_2_O_6_, two 2CaO·SiO_2_ silicates, and a tricalcium 3CaO·SiO_2_ silicate. All phase contents determined by XRD are accurate to within ±2–3 wt.% (based on Rietveld refinement and triplicate measurements).

### 3.4. The Effect of Reduction Degree on Softening

As illustrated in Figure 5, the start and end softening temperatures of sinter with varying degrees of basicity are contingent on the extent of reduction. Error bars for softening temperatures are ±10 °C; for reduction degree—±2%. For all compositions that were analyzed, a characteristic minimum was observed in the 40–60% reduction range. Consequently, for a sinter with a basicity of 2.0, the softening start temperature decreases from 1220 °C (0% reduction) to 1055 °C (50% reduction), and the softening end temperature decreases from 1390 °C to 1195 °C. The softening interval is reduced from 170 to 140 °C.

The underlying cause of this phenomenon is attributed to the maximum accumulation of wustite during the specified reduction interval. Wustite forms low-melting eutectics with calcium silicates (for example, in the FeO–CaO–SiO_2_ system, the eutectic temperature is approximately 1150 °C), which leads to the early appearance of the liquid phase and shrinkage of the material [18,19,22]. Further reduction (exceeding 60%) results in a decrease in the amount of wustite, leading to the dominance of the metallic phase, which possesses a higher melting point. This shift in the softening zone towards higher temperatures is indicative of a phase transition.

It is important to note that the effect of basicity on softening temperatures at a reduction degree of 40–60% is negated: for all samples (basicity 1.2–3.0), the values of T_start_ are 1040–1065 °C, and T_end_—1170–1210 °C. This finding suggests that the phase composition of the material, primarily the presence of wustite, is the determining factor in its behavior within this critical range, rather than the initial basicity.

### 3.5. The Mechanism of Cohesion Zone Formation

The experimental results allow a concise description of the cohesion zone formation. When the sinter reaches a reduction degree of 40–60%, wustite accumulates and forms low-melting eutectics with calcium silicates (e.g., in the FeO–CaO–SiO_2_ system, eutectic temperature 1150 °C). This leads to intensive softening and melting, creating a wide cohesion zone. If the sinter is pre-reduced above 60%, metallic iron dominates, which has a much higher solidus temperature. Consequently, the softening temperatures rise and the cohesion zone narrows, improving gas permeability. The initial basicity has a negligible effect in the 40–60% reduction range, but at reduction > 60%, highly basic sinter (2.0–2.5) maintains higher softening temperatures, which is beneficial for blast furnace performance.

## 4. Conclusions

The present study examined sinter with a basicity ranging from 1.2 to 3.0. The main findings are as follows.

As the basicity increases, the content of the SFCA binder phase rises from 6.2 to 17.5 wt.% and the hematite proportion decreases. The start and end temperatures of softening increase, and the softening interval narrows. From the perspective of high-temperature properties, an optimal basicity range is 2.0–2.5.

During sinter reduction, a sequential phase transformation occurs: magnetite and SFCA → wustite + silicates → metallic iron + silicates. The maximum accumulation of wustite is achieved at a reduction degree of 40–60%, irrespective of basicity.

In the 40–60% reduction range, the lowest softening start (1040–1065 °C) and end (1170–1210 °C) temperatures are observed for all studied sinters. This is attributed to the formation of low-melting eutectics in the FeO–CaO–SiO_2_ system. Within this range, the effect of initial basicity becomes negligible.

To improve blast furnace gas dynamics, the cohesion zone should be lowered. This requires that the sinter reaches a reduction degree of at least 60% before entering the softening zone. Under such conditions, sinter with a basicity of 2.0–2.5 is preferable because it maintains higher softening temperatures after deep reduction.

The quantitative data on phase transformations and their relationship to softening temperature ranges can be used to refine mathematical models of the blast furnace process and to optimize the charge composition.

During reduction and softening above 1100 °C, a calcium–alumina–silicate liquid phase forms in the sinter. Upon cooling, this liquid partially solidifies as an amorphous (glassy) phase. Both the transient liquid and the residual glass influence the mechanical properties and softening behavior, alongside the crystalline phases discussed above.

## Figures and Tables

**Table 1:** Chemical composition of sinter, wt.%.

Basicity	Fe_total_	FeO	CaO	SiO_2_	MgO	Al_2_O_3_	MnO
1.2	61.15	12.38	5.94	4.95	1.77	0.55	0.31
1.4	60.00	10.23	6.96	4.96	2.37	0.57	0.40
1.6	59.09	12.07	8.08	5.05	2.40	0.62	0.41
1.8	58.39	12.37	9.08	5.04	2.40	0.63	0.29
2.0	57.65	8.35	10.11	5.06	2.40	0.64	0.33
2.5	55.44	10.95	12.73	5.05	2.40	0.63	0.29
3.0	53.27	9.49	15.35	5.05	2.40	0.64	0.40

Note: the sum of major oxides does not equal 100% due to the presence of unlisted impurities (P_2_O_5_, TiO_2_, S, alkalis, etc.) and uncertainties of chemical analysis.

**Table 2:** Phase composition of the initial sinter based on X-ray diffraction analysis (wt.%).

Basicity	Fe_3_O_4_	Fe_2_O_3_	β-2CaO·SiO_2_	α-, γ-2CaO·SiO_2_	SFCA
1.2	73.58	12.56	4.28	2.76	6.16
1.4	73.26	11.35	3.37	1.88	8.80
1.6	75.50	4.43	6.14	1.54	9.10
1.8	74.72	6.13	6.34	1.00	11.76
2.0	75.24	6.51	6.47	0.00	12.47
2.5	76.28	2.57	7.07	2.52	14.30
3.0	76.20	2.27	7.12	1.42	17.50

## Data Availability

The original contributions presented in the study are included in the article, further inquiries can be directed to the corresponding author.
